# Association between *CD14* Gene Polymorphisms and Cancer Risk: A Meta-Analysis

**DOI:** 10.1371/journal.pone.0100122

**Published:** 2014-06-30

**Authors:** Jun Wang, Xufeng Guo, Shijie Yu, Jia Song, Jixiang Zhang, Zhuo Cao, Jing Wang, Min Liu, Weiguo Dong

**Affiliations:** Department of Gastroenterology, Renmin Hospital of Wuhan University, Wuhan, Hubei Province, People's Republic of China; Sanjay Gandhi Medical Institute, India

## Abstract

**Background:**

Two polymorphisms, -260C/T and -651C/T, in the *CD14* gene have been implicated in susceptibility to cancer. However, the results remain inconclusive. This meta-analysis aimed to investigate the association between the two polymorphisms and risk of cancer.

**Methods:**

All eligible case-control studies published up to March 2014 were identified by searching PubMed, Web of Science, CNKI and WanFang database. Pooled odds ratio (OR) with 95% confidence interval (CI) were used to access the strength of this association in fixed- or random-effects model.

**Results:**

17 case-control studies from fourteen articles were included. Of those, there were 17 studies (4198 cases and 4194 controls) for -260C/T polymorphism and three studies (832 cases and 1190 controls) for -651C/T polymorphism. Overall, no significant associations between the two polymorphisms of *CD14* gene and cancer risk were found. When stratified by ethnicity, cancer type and source of control, similar results were observed among them. In addition, in further subgroups analysis by *Helicobacter pylori* (*H. pylori*) infection status and tumor location in gastric cancer subgroup, we found that the *CD14* -260C/T polymorphism may increase the risk of gastric cancer in *H. pylori*-infected individuals.

**Conclusions:**

This meta-analysis suggests that the *CD14* -260C/T polymorphism may increase the risk of gastric cancer in *H. pylori*-infected individuals. However, large and well-designed studies are warranted to validate our findings.

## Introduction

Cancer is a major public health problem worldwide and about 12.7 million cancer cases and 7.6 million cancer deaths were reported based on GLOBOCAN 2008 [1]. It is well known that cancer is a multistep process resulting from complex interactions between genetic and environmental factors [2], [3]. Despite the latter play important roles in the development of cancer. Host genetic factors are closely related to the pathophysiology of many human cancers [4]. Variants in several innate immunity genes have been identified as biologically plausible candidates for effects on cancer, such as *CD14*.

The *CD14* gene is localized on chromosome 5q31.1, which encodes a receptor protein that binds to lipopolysaccharide (LPS), its primary ligand, and interacts with co-receptors toll-like receptor 4 (*TLR4*) and lymphocyte antigen 96 (LY96) [5], [6]. CD14 is expressed on the surface of monocytes, macrophages, and neutrophils as membrane CD14 (mCD14) and in the serum as soluble CD14 (sCD14) and its expression may be partially regulated at the genetic level [7], [8]. There are several polymorphism sites in the *CD14* gene, and two well-studied common SNPs in the promoter region of *CD14*, -260C/T (rs2569190; also reported as *CD14* -159) and -561C/T (rs5744455), are investigated extensively to the susceptibility of cancer [9]–[27]. However, the results remain controversial. In this study, we conduct a meta-analysis to evaluate the association between the two polymorphisms and cancer risk.

## Materials and Methods

### Search strategy

We searched the PubMed, Web of Science, CNKI and WanFang database before March 1, 2014, by using the key subjects “cancer”, “carcinoma”, “genetic polymorphism”, “polymorphism”, “variant” in combination with “cluster of differentiation 14”, “CD14”. Additional studies were identified by a hand search of references of original or review articles on this topic. Search results were restricted to human populations and articles were written in English or Chinese. If more than one geographic or cancer type was reported in one report, each was extracted separately. If data or data subsets were published in more than one article, only the publication with the largest sample size was included.

### Inclusion criteria and exclusion criteria

Studies were included according to the following criteria: (1) studies that evaluated the association between the *CD14* polymorphisms and cancer, (2) designed in case-control study, and (3) detailed genotype frequency of cases and controls were provided directly or could be calculated from the article text. Studies were excluded when they were: (1) case-only study, case reports, and review articles, (2) studies without the raw data of the -260C/T genotype of *CD14*, (3) repetitive publications, and (4) studies deviated from the Hardy-Weinberg equilibrium (HWE), (5) animal studies.

### Data extraction

For each study, the following data were extracted independently by two investigators: the first author's name, year of publication, country of origin, ethnicity of study population, cancer type, source of control, genotype method, number of cases and controls and HWE in controls (*P* value). The results were compared, and disagreements were discussed among all authors and resolved with consensus.

### Statistical analysis

The risk of cancer associated with the *CD14* polymorphisms was estimated for each study by odds ratio (OR) and 95% confidence interval (CI). Four different ORs were calculated: dominant model (CT+TT vs. CC), recessive model (TT vs. CT+CC), heterozygote comparison (CT vs. CC), and homozygote comparison (TT vs. CC). A χ^2^-test-based Q statistic test was performed to assess the between-study heterogeneity [28]. When a significant Q test (*P*>0.1) indicated homogeneity across studies, the fixed effects model was used [29], otherwise, the random effects model was applied [30]. We also quantified the effect of heterogeneity by *I* ^2^ test (*I* ^2^<25%: no heterogeneity; *I* ^2^ = 25–50%: moderate heterogeneity; *I* ^2^ = 50–75%: large heterogeneity, *I* ^2^>75%: extreme heterogeneity) [31]. HWE among controls for each study was examined by χ^2^ test. We performed stratification analyses on ethnicity, tumor type and source of control. If any cancer type less than three studies was combined into “other” cancers. Additionally, we also conducted subgroup analysis by *H. pylori* infection status and tumor location in gastric cancer group. Analysis of sensitivity was performed to evaluate the stability of the results, namely, a single study in the meta-analysis was deleted each time to reflect the influence of the individual data set to the pooled OR. Finally, potential publication bias was investigated using Begg' funnel plot and Egger's regression test [32], [33]. *P*<0.05 was regarded as statistically significant.

All statistical analyses were performed using the Cochrane Collaboration RevMan 5.2 and STATA package version 12.0 (Stata Corporation, College Station, Texas).

## Results

### Study characteristics

**Figure 1:**
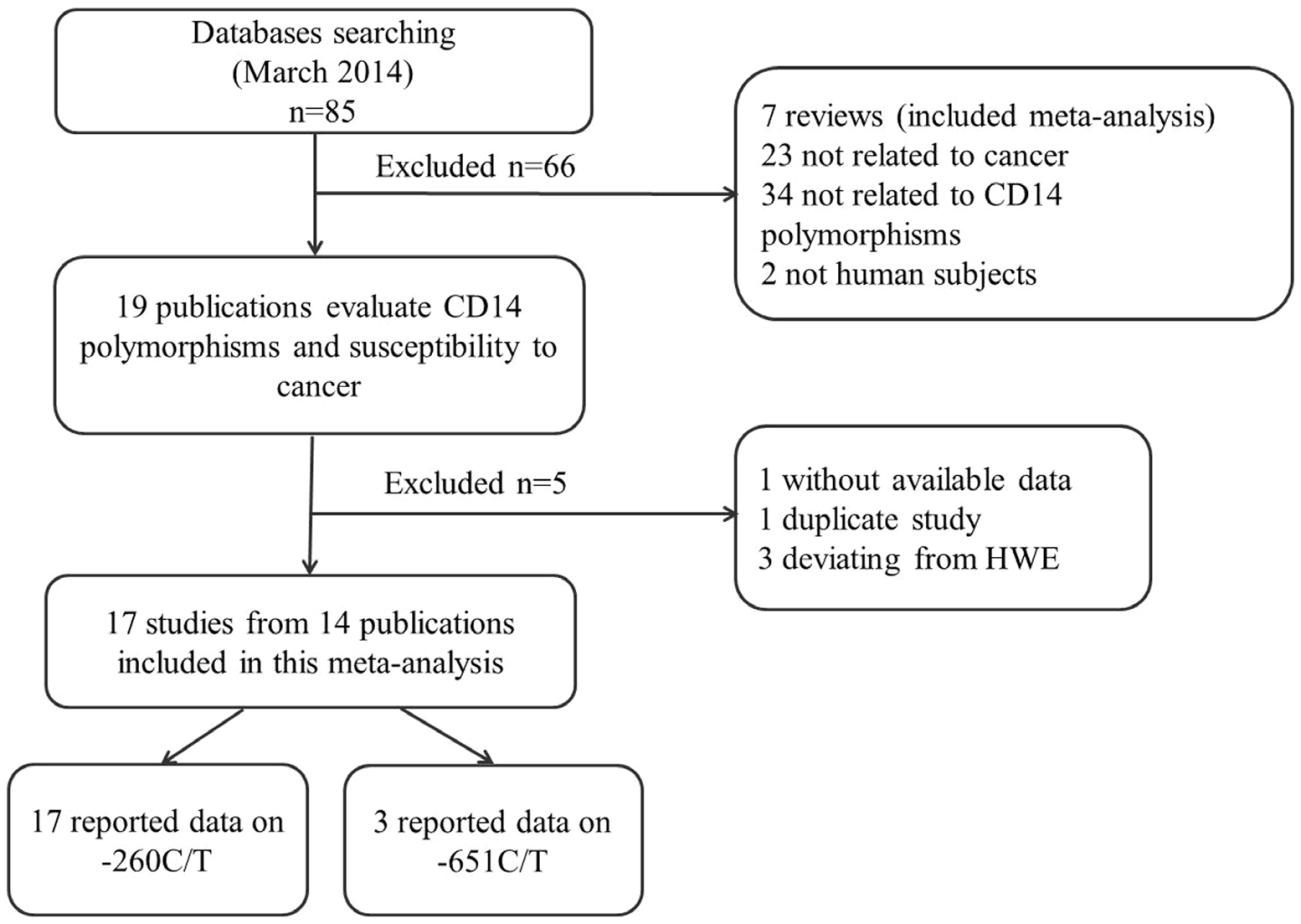
Flow chart showing study selection procedure.

Following the searching strategy, 85 potentially relevant studies were retrieved. According to the inclusion criteria, 19 publications [9]–[27] with full-text were selected and were subjected to further examination. Because the studies [14], [19] included two tumor types respectively and the study by Hold et al [14] included two populations, we treated them separately in this meta-analysis. We excluded one study because they did not present detailed genotyping information [23]. We also excluded one study [24] because it included the overlapped data with those included in the analysis [12]. Furthermore, we removed 3 studies because their genotype distributions among the controls deviated from HWE [25]–[27]. The flow chart of study selection in summarized in Figure 1. As shown in Table 1, therefore, a total of 17 studies from 14 publications were included. Of those, there were 17 studies with 4198 cases and 4194 controls concerning -260C/T polymorphism and three studies with 832 cases and 1190 controls concerning -651C/T polymorphism. Among 17 case-control studies, ten studies were conducted in Asians and seven in Caucasians. Two cancer types were addressed: nine studies on gastric and eight on other cancers (2 on colorectal, acute lymphoblastic leukemia (ALL), lymphomas and one on esophageal, prostate, separately).

**Table 1:** Characteristics of studies included in the meta-analysis.

Study	Year	Country	Ethnicity	Cancer type	Source of controls	Genotype methods	Genotype (case/control)				HWE (*P* value)
							Total	CC	CT	TT	
**-260C/T**											
Andrie [9]	2009	USA	Caucasian	Childhood lymphomas	HB	PCR	83/83	31/18	39/42	13/23	0.886
Castano-Rodriguez [10]	2013	Malaysia, Singapore	Asian	Gastric	HB	Real time-PCR	70/214	18/34	38/108	14/72	0.537
Companioni [11]	2013	Mixed	Caucasian	Gastric	PB	Illumina Beadstation	1192/352	307/103	621/173	264/76	0.833
Guo [12]	2006	China	Asian	Colorectal	PB	PCR-RFLP	110/160	35/25	34/77	41/58	0.947
Hao [13]	2010	China	Asian	Gastric	HB	PCR-RFLP	90/100	7/18	45/52	38/30	0.581
Hold [14]	2009a	Polish	Caucasian	Gastric	PB	TaqMan	327/389	110/131	134/176	83/82	0.112
Hold [14]	2009b	USA	Caucasian	Gastric	PB	TaqMan	306/211	91/52	147/108	68/51	0.730
Hold [14]	2009c	USA	Caucasian	Esophageal	PB	TaqMan	158/211	50/52	74/108	34/51	0.730
Landi [15]	2006	Spain	Caucasian	Colorectal	HB	TaqMan	281/265	62/65	151/137	68/63	0.580
Miedema [16]	2012	Nertherland	Caucasian	Childhood ALL	PB	PCR	186/182	46/28	81/101	59/53	0.077
Min [17]	2012	China	Asian	Prostate	HB	PCR-LDR	168/208	73/102	71/80	24/26	0.105
Tahara [18]	2007	Japan	Asian	Gastric	HB	PCR-RFLP	149/94	37/14	80/53	32/27	0.147
Wu [19]	2006a	China	Asian	Gastric	HB	PCR	204/210	52/54	102/102	50/54	0.679
Wu [19]	2006b	China	Asian	MALT lymphomas	HB	PCR	70/210	17/54	29/102	24/54	0.679
Yu [20]	2011	China	Asian	ALL	PB	PCR-RFLP	174/539	29/80	55/259	90/200	0.796
Zhang [21]	2011	China	Asian	Gastric	HB	PCR-RFLP	160/296	85/141	61/135	14/20	0.102
Zhao [22]	2007	China	Asian	Gastric	PB	PCR-RFLP	470/470	33/56	225/227	212/187	0.305
**-651C/T**											
Miedema [16]	2012	Nertherland	Caucasian	ALL	PB	PCR	188/181	108/96	66/77	14/8	0.124
Yu [19]	2011	China	Asian	ALL	PB	PCR-RFLP	174/539	99/287	60/213	15/39	0.952
Zhao [22]	2007	China	Asian	Gastric	PB	PCR-RFLP	470/470	257/257	191/183	22/30	0.735

HWE: Hardy-Weinberg equilibrium; PB: population-based; HB: hospital-based; PCR-RFLP: polymerase chain reaction-restriction fragment length polymorphism; PCR-LDR: polymerase chain reaction-ligase detection reaction;

ALL: acute lymphoblastic leukemia; MALT lymphomas: gastric mucosa-associated lymphoid tissue lymphoma.

### Quantitative data synthesis

**Figure 2:**
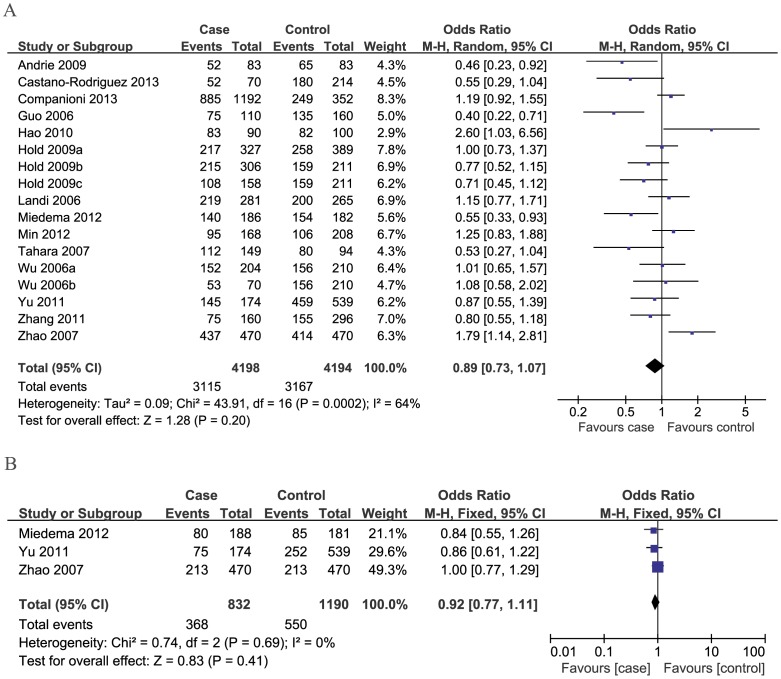
Meta-analysis of the association between *CD14* polymorphisms and susceptibility to cancer under dominant model. A: -260C/T; B: -651C/T.

For 260C/T polymorphism, overall, no significant associations between the *CD14* -260C/T polymorphism and cancer risk were found (dominant model: OR = 0.89, 95%CI: 0.73–1.07; recessive model: OR = 1.08, 95%CI: 0.93–1.25; CT vs. CC: OR = 0.85, 95%CI: 0.70–1.03; TT vs. CC: OR = 0.95, 95%CI: 0.76–1.19) (Table 2, Figure 2A).

**Table 2:** Summary of ORs of the *CD14* polymorphisms and cancer risk.

Variables	n^a^	dominant model			recessive model			CT vs. CC			TT vs. CC		
		OR(95% CI)	*P* b	*I* ^2^	OR(95% CI)	*P* b	*I* ^2^	OR(95% CI)	*P* b	*I* ^2^	OR(95% CI)	*P* b	*I* ^2^
**-260C/T**													
Total	17	0.89(0.73,1.07)	0.0002	64	1.08(0.93,1.25)	0.03	44	0.85(0.70,1.03)	0.0004	62	0.95(0.76,1.19)	0.0005	61
**Ethnicity**													
Asian	10	0.92(0.68,1.25)	0.0006	69	1.15(0.91,1.45)	0.02	55	0.86(0.62,1.17)	0.0008	68	1.03(0.71,1.49)	0.0008	68
Caucasian	7	0.85(0.67,1.08)	0.02	59	1.01(0.87,1.18)	0.39	4	0.84(0.66,1.08)	0.04	55	0.88(0.67,1.15)	0.07	49
**Cancer type**													
Gastric	9	0.99(0.77,1.26)	0.005	63	1.04(0.86,1.26)	0.09	42	0.98(0.78,1.22)	0.03	52	1.03(0.75,1.43)	0.002	67
Others	8	0.78(0.58,1.04)	0.009	63	1.12(0.87,1.44)	0.05	51	0.70(0.51,0.97)	0.005	65	0.86(0.63,1.18)	0.04	53
**Source of control**													
PB	8	0.86(0.65,1.14)	0.0005	73	1.18(1.04,1.33)	0.15	34	0.78(0.56,1.07)	0.0001	76	0.98(0.75,1.30)	0.01	61
HB	9	0.91(0.70,1.19)	0.02	55	0.97(0.74,1.27)	0.05	48	0.93(0.78,1.12)	0.15	34	0.91(0.61,1.36)	0.004	65
**-651C/T**													
Total	3	0.92(0.77,1.11)	0.69	0	1.02(0.70,1.49)	0.21	36	0.92(0.76,1.11)	0.37	0	0.98(0.67,1.44)	0.35	5

a Number of comparison,

b Test for heterogeneity.

In the subgroup analysis on ethnicity, similar results were observed in both Asian and Caucasian populations in all genetic models; when stratified by cancer type, we also failed to detect any association between the -260C/T polymorphism and gastric and other cancers (Table 2).

Stratification based on the source of controls showed significant associations between the -260C/T polymorphism and risk of cancer in the population-based subgroup under recessive model (OR = 1.18, 95%CI: 1.04–1.33). However, no significant association was found in the other three models and population-based subgroup (Table 2).

**Figure 3:**
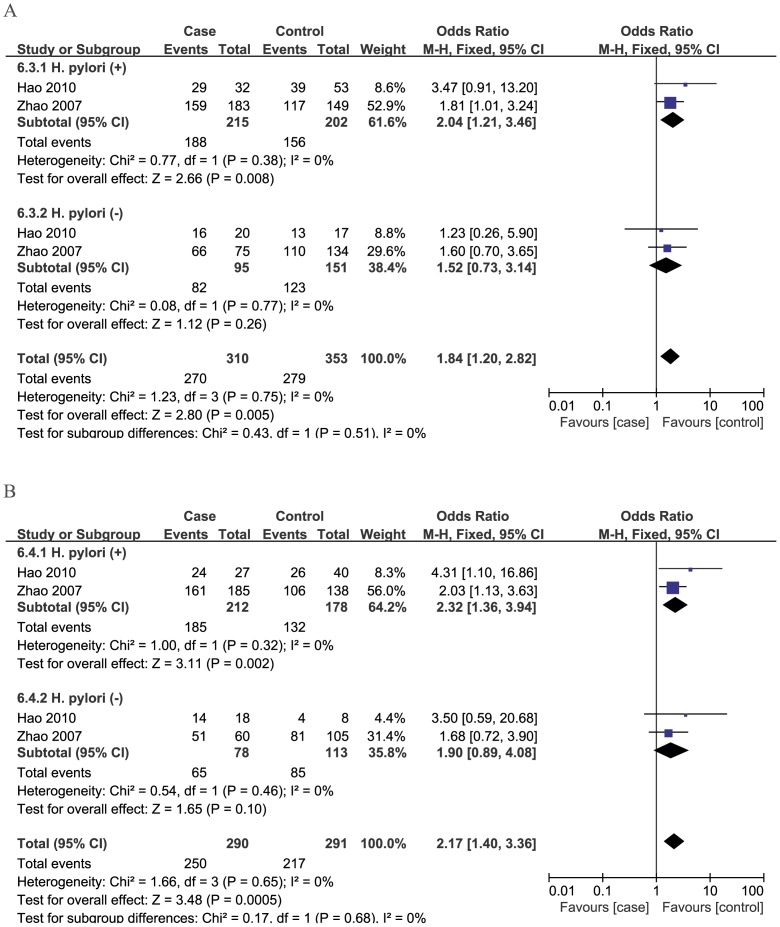
Subgroup analysis by *H. pylori* infection status of odds ratios for association between *CD14* -260C/T polymorphism and risk of gastric cancer. A: CT vs CC; B: TT vs CC.

In addition, in the gastric cancer subgroup, a further stratified analysis based on *H. pylori* infection status and tumor location was conducted. When the analysis was stratified by *H. pylori* infection status, three studies [10], [13], [22] reported the available data and the pooled results showed that the -260C/T polymorphism may be a risk factor for gastric cancer in *H. pylori*-infected individuals (CT vs. CC: OR = 2.04, 95%CI: 1.21–3.46, TT vs. CC: OR = 2.32, 95%CI: 1.36–3.94) (Figure 3). However, in stratified analysis by tumor location, three studies [11], [14], [18] reported the available data and we found that no significant association between -260C/T polymorphism and risk of cardia and non-cardia cancers (Table 3).

**Table 3:** Summary of ORs of the -260C/T polymorphism and gastric cancer risk by *H. pylori* infection status and location.

Variables	n^a^	dominant model			recessive model			CT vs. CC			TT vs. CC		
		OR(95% CI)	*P* b	*I* ^2^	OR(95% CI)	*P* b	*I* ^2^	OR(95% CI)	*P* b	*I* ^2^	OR(95% CI)	*P* b	*I* ^2^
***H. pylori*** ** infection**													
*H. pylori* (+)	3/2*	1.51(0.58,3.92)	0.01	76	1.28(0.95,1.73)	0.59	0	2.04(1.21,3.46)	0.38	0	2.32(1.36,3.94)	0.32	0
*H. pylori* (−)	3/2*	1.44(0.75,2.77)	0.51	0	1.26(0.83,1.93)	0.16	49	1.52[0. 73,3.14]	0.77	0	1.90(0.89,4.08)	0.46	0
**Location**													
Cardia	3	0.75(0.54,1.04)	0.67	0	0.84(0.58,1.22)	0.33	11	0.77(0.54,1.10)	0.42	0	0.69(0.44,1.06)	0.68	0
Non-cardia	3	0.83(0.63,1.08)	0.33	9	0.90(0.68,1.19)	0.25	28	0.83(0.62,1.11)	0.56	0	0.80(0.57,1.13)	0.15	48

a Number of comparison,

b Test for heterogeneity,

* 3 studies in the dominant model, 2 in the other models.

For -651C/T polymorphism, three studies were included. We found no statistical association between the -651 polymorphism and overall cancer risk in all genetic models (Table 2, Figure 2B).

### Heterogeneity and sensitivity analyses

Substantial heterogeneities were observed among studies for the association between the *CD14* -260C/T polymorphism and cancer risk under all genetic models (dominant model: I^2^ = 64%, *P* = 0.0002; recessive model: I^2^ = 44%, *P* = 0.003; CT vs. CC: I^2^ = 62%, *P* = 0.0004; TT vs. CC: I^2^ = 61%, *P* = 0.0005). Then, we assessed the source of heterogeneity for all genetic model comparison by ethnicity, cancer type and source of control. The heterogeneity was partly decreased in Caucasians and hospital-based populations in some models. However, there was still significant heterogeneity among Asians, gastric, population-based and other cancers. Then sensitivity analysis was performed by excluding each study individually to evaluate the stability of the results. The statistical significance of the results was not altered when any single study was omitted, confirming the stability of the results.

### Publication bias

**Figure 4:**
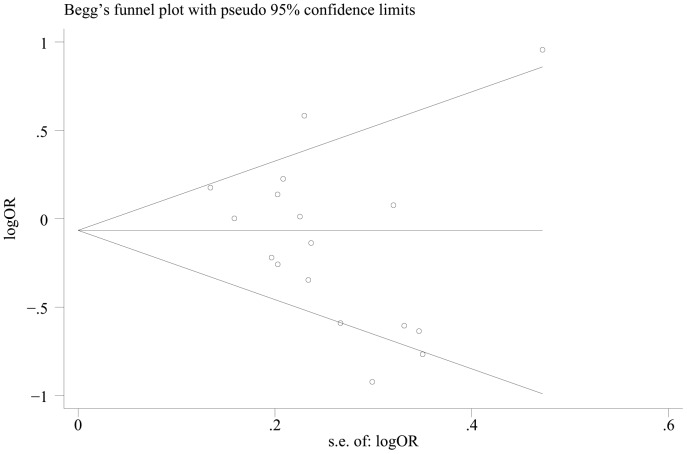
Begg's funnel plot for publication bias (dominant model).

Begg's funnel plot and Egger's test were performed to assess the potential publication bias in the available literature. The shape of funnel plots did not reveal any evidence of funnel plot asymmetry (Figure 4). Egger's test also showed that there was no statistical significance for the evaluation of publication bias (dominant model: *P* = 0.144, CT vs. CC: *P* = 0.117, TT vs. CC: *P* = 0.141, recessive model: *P* = 0.123).

## Discussion

Genetic polymorphisms in genes whose products regulate the immune and antitumor responses in malignancies are good candidates for investigation. Many candidate genes were reported to be associated to cancer risk, such as *TLRs*, *CD14*. TLRs are pattern recognition receptors (PRR) of the innate immune system that recognise a wide variety of molecules. With respect to CD14, it is a pattern-recognition receptor that plays a central role in innate immunity and directs the adaptive immune responses [34]. As a co-receptor of TLRs, CD14 acts primarily by transferring LPS and other bacterial ligands from circulating LPS-binding protein to the TLR4/MD-2 signaling complex. Two common promoter polymorphisms have been identified in the *CD14* gene at positions -260 and -651 from the AUG start codon, which correspond to -159 and -550 designated according to the transcription start site, respectively [35], [36]. With regard to -260C/T polymorphism, LeVan et al. [37] showed that the T allele has a decreased affinity for DNA/protein interactions at a GC box containing a binding site for SP1, SP2, and SP3 transcription factors and leads to an increased transcriptional activity. Consistently, Hartel et al. [38] reported that after in vitro stimulation of cord blood cultures with LPS, carriers of the -159T allele have higher levels of sCD14 compared with carriers of the -159C allele. Recently, the -260C/T polymorphism in *CD14* gene has been investigated the association with many diseases, such as inflammatory bowel disease [39], alcoholic liver disease [40], tuberculosis [41], sepsis [42], coronary heart disease [43], asthma [44] and allergic rhinitis [45]. As for cancer, a previous meta-analysis conducted by Zhou et al. [46], evaluated the association between *CD14* -260C/T polymorphism and risk of cancer based on 12 studies including 2498 cases and 2696 controls and reported that the *CD14* -159C/T gene polymorphism is not a genetic risk factor for cancer.

In this study, we conducted a comprehensive literature search in different databases and included several additional studies, which allowed for a larger number of subjects (17 studies including 4198 cases and 4194 controls) and more precise risk estimation. Besides, we conducted a further stratified analysis based on *H. pylori* infection status and tumor location in gastric cancer group. In addition, we also explore the association between *CD14* -651C/T polymorphism and risk of cancer based on three studies with 832 cases and 1190 controls. The pooled data demonstrated that no significant associations between the two polymorphisms of *CD14* gene and cancer risk were found in overall comparison. Besides, in the subgroup analysis by ethnicity and cancer type, we also failed to detect any association between the -260C/T polymorphism and risk of Asians, Caucasians, gastric and other cancers. However, when stratified by source of control, a significant association between the -260C/T polymorphism and risk of cancer in the population-based subgroup was found under recessive model. The results seem to contradict the observations of functional studies of CD14, which had suggested that CD14 played an important role in the development of cancer. Since carcinogenesis is a multistep process involving multifactorial interplay between genetic and environmental factors that involves various genetic alterations and several biological pathways. Thus, it is unlikely that risk factors of cancer work in isolation from each other. What's more, the different linkage disequilibrium patterns usually exist in related genes and the influence of the genetic variant may be masked by other unidentified causal genes involved in carcinogenesis. In addition, only few studies on -651C/T polymorphism were included, which may also contribute to the result and it should be interpreted with caution.

As *H. pylori* infection is known to be the main risk factor for gastric cancer [47], we examined the potential interaction between *H. pylori* infection and CD14 -260C/T polymorphism in the development of gastric cancer. The pooled results showed that the -260C/T polymorphism may be a risk factor for gastric cancer in *H. pylori*-infected individuals. Since mCD14 is mostly expressed in monocytes/macrophages, which are accumulated in *H. pylori* infected mucosa [48]. That is, individual with CT/TT genotype had higher sCD14 levels compared with the carriers with C allele. The results indicate that -260C/T polymorphism might play a role in the outcome of *H. pylori* infection, especially the development of gastric cancer. In addition, we also explored the -260C/T polymorphism association with both anatomical localizations of gastric cancer and there was no significant association between -260C/T polymorphism and risk of cardia and non-cardia cancers. However, because only few studies were included in the above analysis, the result should be interpreted with caution, and more studies are needed.

Heterogeneity is a potential problem when interpreting the results of all meta-analysis [49]. In this meta-analysis, heterogeneity was found in overall comparison in three genetic models, when stratified by ethnicity, cancer type and source of control, the heterogeneity was partly decreased in Caucasians and hospital-based populations. However, heterogeneity still existed among Asians, population-based, gastric and other cancers. Then sensitivity analyses were conducted by successively excluding one study, the estimated pooled odd ratio changed quite little, strengthening the results from this meta-analysis. The results above suggest that the different ethnicities, cancer type and population selection might contribute to the heterogeneity observed in the meta-analysis. Besides, lifestyle, environmental background and other unknown factors may also be the source of heterogeneity. No publication bias was shown suggesting this possible true result.

In interpreting our results of the current meta-analysis, some limitations should be acknowledged. First, the controls were not uniformly defined. Some studies used a healthy population as the control group, whereas others selected patients without cancers in hospital as the reference group. Therefore, the controls may not always be truly representative in the underlying source populations, especially when the polymorphism is also expected to affect the risk of other diseases. Second, the number of published studies was not sufficiently large for a comprehensive analysis, particularly for subgroup analysis by cancer type. Thus, we may fail to explore the real association between the polymorphism and specific cancer type (such as colorectal, ALL). Third, because of the lack of original data, our results were based on single-factor estimates without adjustment for age, gender and other risk factors (e.g. smoking, drinking status), which may cause serious confounding bias.

In conclusion, this meta-analysis suggests that the *CD14* -260C/T polymorphism may increase the risk of gastric cancer in *H. pylori*-infected individuals. However, large and well-designed studies are warranted to validate our findings. Moreover, more gene-gene and gene-environment interactions should also be considered in future analysis, which should lead to better, comprehensive understanding of the association between the *CD14* polymorphisms and cancer risk.

## Supporting Information

Checklist S1 **PRISMA Checklist.**
(DOC)

Checklist S2 **MOOSE Checklist.**
(DOC)
