# Supplementary material for: Association between CD14 Gene Polymorphisms and Cancer Risk: A Meta-Analysis
Source: PLoS One. 2014 Jun 30;9(6):e100122. doi: 10.1371/journal.pone.0100122 (PMC4076245; doi:10.1371/journal.pone.0100122)
Supplement: Checklist S2 — MOOSE Checklist. (DOC) [file pone.0100122.s002.doc]

**MOOSE Checklist**

1. **Reporting of background should include**

**Problem definition:** The CD14 polymorphisms and cancer risk

**Hypothesis statement:** CD14 polymorphism is not related to cancer risk

**Description of study outcome:** The two polymorphisms (-260C/T and -651C/T) of CD14 gene are not associated with the risk of cancer.

**Type of exposure or intervention used:** CD14 polymorphisms

**Type of study designs used:** Meta-analysis

**Study population:** Cancer patients and healthy controls

1. **Reporting of search strategy should include**

**Qualifications of searchers:** Wang J and Guo XF

**Search strategy, including time period include in the synthesis and keywords:**

PubMed from 1965 –March 2014

Web of Science from 1970 –March 2014

CD14; polymorphism; cancer

**Effort to include all available studies, including contact with authors:** Yes

**Databases and registries searched:** PubMed, Web of Science, CNKI, WanFang

**Search software used, name and version, including special features used:** We did not employ any search software. EndNote was used to merge retrieved citations and eliminate duplications

**Use of hand searching:** Yes

**List of citations located and those excluded, including justification:** Figure 1 and Table 1

**Method of addressing articles published in languages other than English:** Translation software

**Method of handing abstracts and unpublished studies:** No unpublished studies were observed.

**Description of any contact with authors:** None

1. **Reporting of methods should include**

**Description of relevance or appropriateness of studies assembled for assessing the hypothesis to be tested:** Table 1

**Rationale for the selection and coding of data:** (1) studies that evaluated the association between the CD14 polymorphisms and cancer, (2) in a case-control study design, and (3) had detailed genotype frequency of cases and controls or could be calculated from the article text.

**Documentation of how data were classified and coded:** Two investigators (Wang and Guo) used a standard protocol and data-collection form. They discussed with Pro. Dong, then decide the data.

**Assessment of confounding:** No restricted for the analysis. We conducted sensitivity analyses by eliminating one study at a time.

**Assessment of study quality, including binding of quality assessors; stratification or regression on possible predictors of study results:** The results of sensitivity analyses were very stable.

**Assessment of heterogeneity:** The chi-square-based *Q*-test and *I^2^* test

**Description of statistical methods in sufficient detail to be replicated:** A χ^2^-test-based Q statistic test was performed to assess the between-study heterogeneity. We also quantified the effect of heterogeneity by *I*^2^ test. When a significant Q test (*P*<0.05) or *I*^2^ >50% indicated heterogeneity across studies, the random effects model was used, or else the fixed effects model was used.

**Provision of appropriate tables and graphics:** We included the terms used for database search, 1 flow chart, 3 summary table, 1 forest plots of all studies,1 funnel plot to examine publish bias.

1. **Reporting of results should include**

**Graphic summarizing individual study estimates and overall estimate:** Table 2, Table 3, Figure 2, Figure 3

**Table giving descriptive information for each study included:** Table 1

**Results of sensitivity testing:** Table 2

**Indication of statistical uncertainty of findings:** 95% confidence intervals were presented with all summary estimates, *P* values and results of sensitivity analyses.

1. **Reporting of discussion should include**

**Quantitative assessment of bias:** Sensitivity analyses indicate this non-significant association was stable.

**Justification for exclusion:** We excluded studies that had used different exposure or outcome assessment for the comparison groups, or no control group.

**Assessment of quality of included studies:** We discussed the results of the sensitivity analyses.

1. **Reporting of conclusions should include**

**Consideration of alternative explanations for observed results:** We discussed that potential unmeasured confounders such as differences of ethnicity, tumor type, the source of controls, life style, environment background and other unknown factors may be the source of heterogeneity.

**Generalization of the conclusions:** The two polymorphisms (-260C/T and -651C/T) of CD14 gene are not associated with the risk of cancer.

**Guidelines for future research:** Future studies should use standardized unbiased genotyping methods and homogeneous cancer patients and well-matched controls and include multiethnic groups.

**Disclosure of funding source:** No funding supported this study.
